# Inducing Stable α + β Microstructures during Selective Laser Melting of Ti-6Al-4V Using Intensified Intrinsic Heat Treatments

**DOI:** 10.3390/ma10030268

**Published:** 2017-03-07

**Authors:** Pere Barriobero-Vila, Joachim Gussone, Jan Haubrich, Stefanie Sandlöbes, Julio Cesar Da Silva, Peter Cloetens, Norbert Schell, Guillermo Requena

**Affiliations:** 1 Institute of Materials Research, German Aerospace Center (DLR), Linder Höhe, Cologne 51147, Germany; joachim.gussone@dlr.de (J.G.); jan.haubrich@dlr.de (J.H.); guillermo.requena@dlr.de (G.R.); 2 Institute of Materials Science and Technology, Vienna University of Technology, Karlsplatz 13/308, Vienna 1040, Austria; 3 Department and Chair of Physical Metallurgy and Metal Physics, RWTH Aachen University, Kopernikusstr.14, Aachen 52074, Germany; sandloebes@imm.rwth-aachen.de; 4 European Synchrotron Radiation Facility (ESRF), Avenue des Martyrs 71, Grenoble 38000, France; jdasilva@esrf.fr (J.C.D.S.); cloetens@esrf.fr (P.C.); 5 Helmholtz-Zentrum Geesthacht, Max-Planck-Straße 1, Geesthacht 21502, Germany; norbert.schell@hzg.de; 6 Metallic Structures and Materials Systems for Aerospace Engineering, RWTH Aachen University, Aachen 52062, Germany

**Keywords:** additive manufacturing, selective laser melting, intrinsic heat treatment, titanium alloys, metastable phases, phase transformations, martensite decomposition, element partitioning, high energy synchrotron X-ray diffraction, synchrotron holographic X-ray computed tomography

## Abstract

Selective laser melting is a promising powder-bed-based additive manufacturing technique for titanium alloys: near net-shaped metallic components can be produced with high resource-efficiency and cost savings. For the most commercialized titanium alloy, namely Ti-6Al-4V, the complicated thermal profile of selective laser melting manufacturing (sharp cycles of steep heating and cooling rates) usually hinders manufacturing of components in a one-step process owing to the formation of brittle martensitic microstructures unsuitable for structural applications. In this work, an intensified intrinsic heat treatment is applied during selective laser melting of Ti-6Al-4V powder using a scanning strategy that combines porosity-optimized processing with a very tight hatch distance. Extensive martensite decomposition providing a uniform, fine lamellar α + β microstructure is obtained along the building direction. Moreover, structural evidence of the formation of the intermetallic α_2_-Ti_3_Al phase is provided. Variations in the lattice parameter of β serve as an indicator of the microstructural degree of stabilization. Interconnected 3D networks of β are generated in regions highly affected by the intensified intrinsic heat treatment applied. The results obtained reflect a contribution towards simultaneous selective laser melting-manufacturing and heat treatment for fabrication of Ti-6Al-4V parts.

## 1. Introduction

The invention of additive manufacturing (AM) methods such as selective laser melting (SLM) is about to represent a paradigm change in the industry. SLM is a powder-bed-based AM technique that allows near net-shape manufacturing of metallic components. One of the main key strengths of SLM is that extremely complex geometries (bionic or load-optimized) can be manufactured: inner channels and cavities (e.g., for cooling fluids), inaccessible using conventional manufacturing techniques, can lead to structures of minimal weight and optimized functional performance. These advantages concisely explain the increasing interest to implement SLM as a regular production method for titanium alloys [1,2].

Metallurgy plays a central role to advancing the understanding of SLM and further metal additive manufacturing methods owing to the complex, currently untunable thermal history that determines the microstructure and, hence, the structural performance of components. This is characterized by a series of sharp thermal cycles tracing fast heating (~10^6^–10^7^ K/s) and cooling rates (~10^3^–10^8^ K/s) which, depending on the material system, can induce the formation of metastable, brittle microstructures with low fatigue resistance, i.e., unsuitable for structural applications [3,4,5,6,7]. Thus, post-thermal and/or thermomechanical treatments for microstructural adjustment are required to provide the mechanical properties needed: a costly methodology that restricts the economical attractiveness of SLM.

Besides steel, the classical α + β Ti-6Al-4V (Ti-64) alloy represents the most investigated SLM-produced material. It is also the most commercialized titanium alloy nowadays, holding more than 50% of the global market of titanium alloys. Ti-64 offers a well-balanced property profile, particularly as a forged product [2,8,9].

Owing to the β → α’ transformation occurring at fast cooling rates, SLM of Ti-64 powders usually results in extensive formation of brittle martensitic microstructures. Previous investigations reported that α’ → α + β martensite decomposition—leading to configurations of α and β phases that render acceptable mechanical performance—can be induced during SLM via intrinsic heat treatment (IHT) by alteration of processing parameters including energy density and temperature of the building platform (e.g., [10,11]). However, modifications of these parameters can also imply the formation of void defects (i.e., round or crack-like pores) and a decrease in the ductility of the alloy via excessive oxygen pick up [12,13]. Thus, exploration of alternative laser scanning strategies based on a compromise between microstructure design and minimization of bulk defects is required to improve the SLM manufacturability of Ti-64.

The present study aims at exploring the capability of intense IHT to produce Ti-64 by SLM using a combination of porosity-optimized processing parameters based in our previous studies [12] with a tight hatch distance. This intensified IHT with longer exposure periods of the alloy to high temperatures should allow simultaneous SLM-fabrication plus decomposition of α’ martensite into stable α and β phases in one step. Conventional and advanced metallographic analysis using high-resolution synchrotron holographic X-ray computed tomography (HXCT) provides 2D as well as 3D sub-µm microstructural information on the alloy. Also, structural and non-destructive bulk evaluation employing high-energy synchrotron X-ray diffraction (HEXRD) allows analysis of the influence of diffusion processes in the stabilization of the alloy along the building direction. The advances obtained will serve to advance towards qualification of Ti-64 SLM parts.

## 2. Materials and Methods

### 2.1. Selective Laser Melting Process

SLM of a Ti-6Al-4V wt % grade 5 powder alloy (max. 0.2 wt % O) was carried out in an argon 5.0 atmosphere employing a SLM250^HL^ machine with constant temperature of the building platform of 200 °C. The SLM equipment as well as the powder alloy produced by gas atomization were supplied by SLM solutions GmbH. The powder consisted of spherical particles with a size distribution according to the following D-values as measured by laser diffraction (Beckman Coulter LS 13320 PIDS): D10 = 30 µm, D50 = 43 µm and D90 = 55 µm. Cubes of 10 × 10 × 10 mm^3^ were built using a zig-zag scanning with an increment of 90° from layer to layer [14]. Although variations in the sample size can alter the resulting SLM-produced microstructures, the chosen dimensions permit evaluation of the effect of different sets of scanning parameters for a batch of specimens in a single machine job. The delay time between two subsequent layers was ~120 s.

The strategy of the SLM process aims to explore the possibilities offered by the intrinsic heat treatment induced by the scanning laser in order to reach configurations of stable α and β phases in the Ti-64 SLM alloy in a single SLM process step. For this, a configuration of optimized processing parameters (see Table 1), i.e., those for which minimum porosity was achieved in a previous study [12], was modified by applying a very tight hatch distance (*h* = 40 µm). The reduced hatch spacing leads to an increased laser exposure time, *t_exp_* (time during which the bulk material is subjected to the temperature influence of the scanning laser) and consequently, to an intensified intrinsic heat treatment of the Ti-64 alloy with longer heating periods. This excludes eventual influence of the cooling rate. The effective exposure time can be approximated as:

(1) $$t_{exp}=\frac{V}{v\cdot x\cdot h}\;\left[s\right],$$

with volume of the sample *V*, scan rate *v*, layer thickness *x* and hatch distance *h*. The exposure time resulting from the reduced hatch distance used is therefore three times longer compared to the usual SLM setups that provide sufficient line-scanning overlapping of molten pools (e.g., *h* ~ 120 µm) [12]. Besides increased re-melting, the energy transferred to underlying layers using this approach increases without risk of overheating, consequent loss of Al and severe formation of keyhole pores [12,15].

### 2.2. Characterization of the Ti-6Al-4V Alloy

#### 2.2.1. Microscopy

Light optical microscopy (LOM) and scanning electron microscopy (SEM) of Ti-64 SLM samples prepared by grinding and polishing—including a 3-µm diamond suspension and SiO_2_-H_2_O-H_2_O_2_ for the last two steps—was carried out employing a ZEISS LSM 700 and a dual beam FEI Helios Nanolab 600i (electron and Ga^+^) setup with an integrated SEM unit, respectively. A solution of H_2_O + 4.5 mol/L KOH + 2.5 mol/L H_2_O_2_ with a soaking time of 80 s was used to etch the samples for LOM examination. SEM analysis was operated in backscattered electron mode (BSE).

Thin-foil specimens were prepared for transmission electron microscopy (TEM) from 3-mm discs by mechanical grinding to a thickness of 100 µm and subsequent electro-polishing using an electrolyte containing 59% methanol, 35% ethylene glycol monobutyl ether and 6% perchloric acid at −20 °C and 25 V. The samples were examined using a Philips CM20 microscope operated at 200 kV.

#### 2.2.2. Hardness

Vickers microhardness measurements of the mirror-polished, unetched central section of the Ti-64 SLM samples were performed along the SLM building direction *z* using a Clemex MMT-X7 tester. Line sequences of indentations covering the complete height of the sample (from *z* = 0 mm to *z* = 10 mm) as well as the local upper surface with higher resolution (from *z* = 9.5 mm to *z* = 10 mm), were carried out using 200 g (HV 0.2) and 50 g (HV 0.05), respectively. The values obtained correspond to an average of three different indentations taken for each *z*. The size of the resulting indentations (~30 and 15 µm for HV 0.2 and HV 0.05, respectively) is considerably larger than that of the microstructural features.

#### 2.2.3. High Energy Synchrotron X-ray Diffraction

High energy synchrotron X-ray diffraction (HEXRD) was carried out in transmission mode at the beamline P07-HEMS [16] at PETRA III (Deutsches Elektronen-Synchrotron, DESY, Hamburg, Germany) using the experimental parameters summarized in Table 2. Patterns of entire Debye–Scherrer rings from the bulk Ti-64 SLM alloy were acquired at a step size of 0.5 mm along the building direction *z*, from *z* = 0 mm to *z* = 10 mm. Samples of 4 × 4 × 10 mm^3^ (*thickness* = 4 mm) were investigated and kept fixed during acquisition. Although the diffraction images are associated with specific *z* positions, it is important to consider that they result from a volume comprised within the slit-aperture size along the z direction. The instrumental parameters for calibration were obtained using LaB_6_ powder standard.

Azimuthal integration of the diffraction images for a 2-Theta range between 2.0° and 5.0° was carried out using the software Fit2D [17], while texture analysis (based on a E-WIMV algorithm) and quantitative evaluation of phase fractions and lattice parameters were determined by Rietveld refinement as implemented in the software MAUD [18]. Cake portions of 10° were considered for the texture analysis. Moreover, calculations of the full width at half maximum (FWHM) were determined by single peak fitting (pseudo-voigt approximation) using the software Topas 4.2 [19].

#### 2.2.4. Synchrotron Holographic X-ray Computed Tomography

A micro-cylinder of 16 µm diameter × 18 µm height was prepared for holographic X-ray computed tomography (HXCT) [20,21] from a representative volume of a microstructural region highly affected by the intrinsic SLM heat treatment (i.e., bottom region with building position *z* ~ 0 mm of the SLM sample) using the Helios Nanolab 600i (Section 2.2.1). Firstly, a platinum disc ~17 µm in diameter was deposited over the cross section of interest of a Ti-64 sample. Thereafter, a cylinder was milled through the thickness of the sample by a gallium ion beam and fixed on a tungsten needle using a lift-out-needle.

HXCT was carried out at the nano-imaging beamline ID16A of the European Synchrotron Research Facility (ESRF), Grenoble, France [22]. Phase contrast was required since the microstructural components of the Ti-64 alloy do not produce sufficient absorption contrast [23]. The sample was illuminated with a magnifying X-ray cone beam of 33.6 keV focused by Kirkpatrick–Baez mirrors using the zoom HXCT approach [20]. Then, 2500 projections with an image size of 2048 × 2048 pixels and a field of view of 20.5 × 20.5 µm^2^ were recorded between 0 and 180° employing a CCD camera at four sample-to-focal-point distances (5.54, 5.72, 6.45 and 7.95 mm) for efficient phase retrieval of the holotomographic reconstruction [23]. The experiments were performed in an ultra-high vacuum atmosphere and the acquisition time was ~1 s/projection resulting from the sum of exposure and read-out times. The holographic phase retrieval [21] at each angle was performed using in-house routing written in the GNU Octave programming environment and the public domain image analysis software ImageJ. The reconstructed volume has a voxel size of 10 × 10 × 10 nm^3^. The tomographic reconstruction was carried out using the filtered back projection (FBP) algorithm [24] implemented in the ESRF software PyHST. Pre-image processing using band pass and diffusion filtering was applied to minimize artefacts critical for segmentation. In addition, 3D segmentation of phases was performed by local threshold based on grey-level distribution after image conversion from 32 to 8 bits.

## 3. Results

### 3.1. Microstructural Evolution along the SLM Building Direction

**Figure 1:**
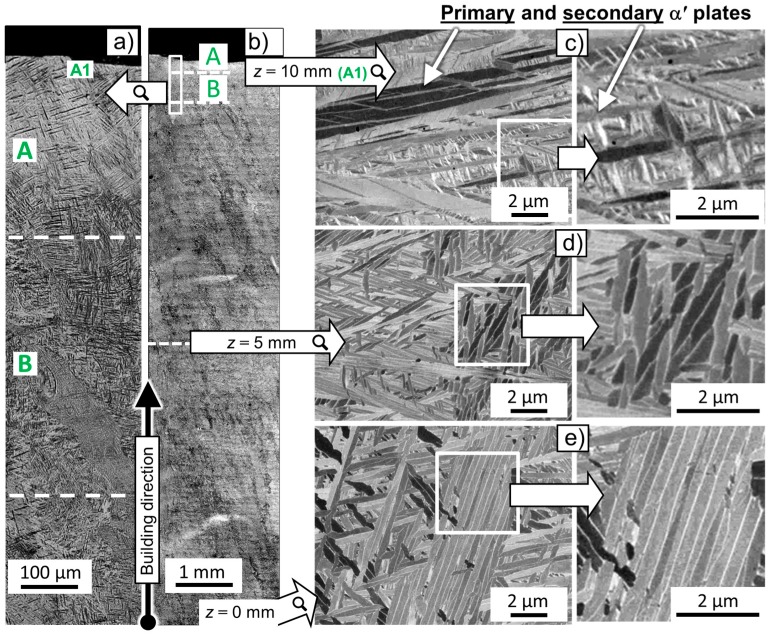
Light optical microscopy (LOM) of (**a**) the upper region and of (**b**) the entire central cross section of a Ti-64 sample along the SLM building direction. Backscattered electron mode-scanning electron microscopy (BSE-SEM) images are shown, corresponding to selected regions of the microstructure for (**c**) *z* ~ 10 mm (top); (**d**) *z* ~ 5 mm (center) and (**e**) *z* ~ 0 mm (bottom).

Figure 1a,b shows LOM micrographs of the upper region and of the complete central cross section of the Ti-64 alloy along the SLM building direction, respectively. The microstructural overview of Figure 1b presents elongated prior β grains formed during the SLM process [25] that are oriented inclined with respect to the building direction of the sample. The distance between grain boundaries in the minor axis direction is ~70–140 µm.

Careful LOM examination of the extreme upper part of the sample (region *A* in Figure 1a) reveals the presence of a top layer of ~150–250 µm with a different etching contrast than the lower bulk alloy (see region *B* of Figure 1a). The BSE image in Figure 1c taken at the top of the sample (*A1*) shows that in this region the interior of prior β grains consists of a hierarchical structure of acicular plates of α’ martensite with a thickness of secondary and primary units with lengths ranging from < 1 µm up to ~30 µm, respectively.

**Figure 2:**
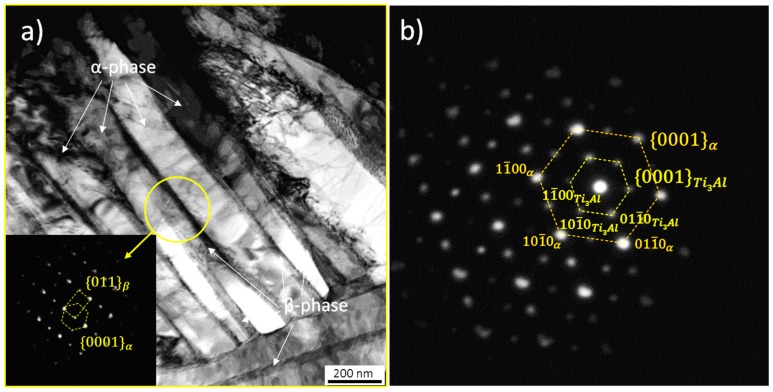
(**a**) Bright field transmission electron microscopy (TEM) image showing the ultrafine α + β microstructure formed at the center of a Ti-64 SLM sample (see *z* ~ 5 mm in Figure 1). The inset shows the Burger’s orientation relationship {011}β // {0001}α between crystal lattices; (**b**) Selected area electron diffraction pattern revealing the presence of α_2_-Ti_3_Al within an α lamella.

In contrast to this predominantly martensitic microstructure in the top layers of the SLM sample, a lamellar α + β microstructure formed by homogeneous packets of fine α lamellae (thickness ~ 100–400 nm) which are separated by thin continuous β layers (i.e., β layers of ~50 nm in thickness), are observed within prior β grains at the center and bottom (see Figure 1d,e) for *z* = 5 mm and *z* = 0 mm, respectively. The BSE observations reveal that the morphological features of this lamellar microstructure remain relatively unaltered along the building direction below the martensitic layer. TEM analysis of this region of the sample revealed that the fine lamellae and inter-lamellae films correspond to α phase and β phases, respectively (Figure 2a). Moreover, a Burger’s orientation relationship (OR) {011}β // {0001}α is obtained between crystal lattices as presented in the selected area diffraction pattern (SADP) in the inset in Figure 2a. SAPD also reveals the presence of α_2_-Ti_3_Al as can be seen in Figure 2b.

**Figure 3:**
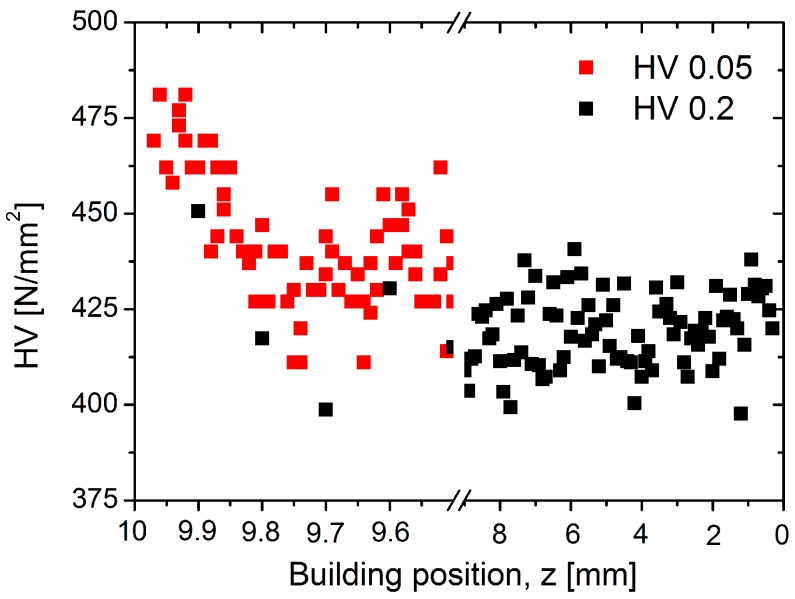
Vickers microhardness as a function of the SLM building position, *z*, with top and bottom of the sample *z* = 0 mm and *z* = 10 mm, respectively (as in Figure 1).

Figure 3 shows that the martensitic layer identified in Figure 1 for the top region of the Ti-64 sample is associated with a steep increase in microhardness of ~50 HV 0.05 between *z* = 9.75 and 10 mm. This range is in agreement with the depth of ~250 µm of the martensitic region observed in Figure 1. In addition to this, a constant mean value of ~420 ± 10 HV 0.2 is obtained for lower building positions of the main bulk of the sample.

**Figure 4:**
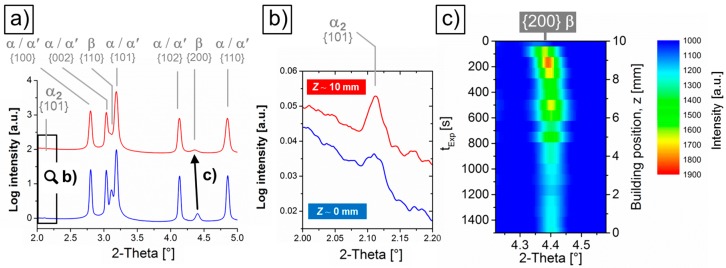
(**a**) Diffraction patterns (limited to a representative 2-Theta range) for the bottom and top building positions of the Ti-64 sample *z* = 0 mm (in blue) and *z* = 10 mm (in red), respectively. {hkl} reflections of α’/α and β are indicated; (**b**) Details of the 2-Theta region selected in (**a**) showing the presence of a low intensity {101} peak of α_2_-Ti_3_Al for both conditions; (**c**) Color-coded 2D intensity plot corresponding to the evolution of the {200} reflection of β (see relative 2-Theta position in (**a**)) as a function of the building position *z* and exposure time *t_exp_*.

Figure 4a shows that the diffraction patterns obtained by HEXRD at the two extremes of the building positions, i.e., *z* = 0 mm (blue) and *z* = 10 mm (red), consist basically of reflections of the hcp and bcc crystal lattices of α/α’ and β, respectively. This is in agreement with the microstructural analysis described in Figure 1 and Figure 2. This technique also confirms the presence of α_2_-Ti_3_Al revealed by TEM (Figure 2b), as can be observed by the {101} reflections of this phase in the 2-Theta detail of Figure 4b.

**Figure 5:**
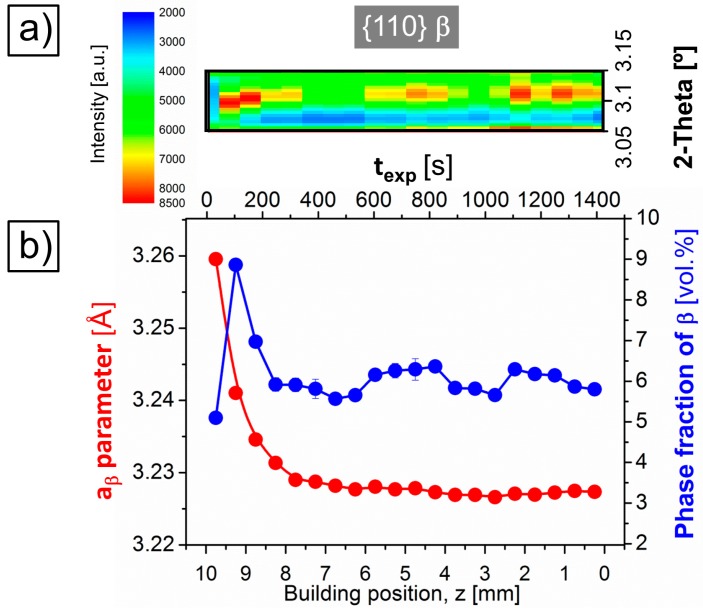
(**a**) Evolution of the intensity of {110} of β (see relative 2-Theta position in the diffractograms of Figure 3a) and (**b**) that of the volume fraction and lattice parameter of β obtained by Rietveld analysis as a function of the building position *z* and exposure time *t_exp_*.

The color-coded 2D intensity plot of Figure 4c shows that no significant 2-Theta variations occur for the {200} reflection of β along the SLM building direction between *z* ~ 0 and 8 mm. Thereafter, in the upper part of the sample a gradual shift of this reflection towards lower 2-Theta angles takes place for higher *z* values. This effect is associated with an expansion of the *d*-spacing of {200} planes or, in other words, with a gradual increase of the lattice parameter of β, *a*_β_, in the upper region of the Ti-64 sample. Accordingly, the quantitative phase analysis of the diffractograms presented in Figure 5b as a function of *z* and *t_exp_* shows that *a*_β_ detaches from linearity (from the constant initial value of 3.229 nm), between *z* = 7.75 and 8.25 mm, and undergoes a steep increase up to 3.259 nm (0.9%) at the top of the sample, *z* = 10 mm. This onset occurs together with a local maximum in the volume fraction of β of 8.8 vol % at *z* = 9.25 mm. As *z* decreases, this parameter decays down to 5.1 vol %, i.e., 0.7 vol % below the initial 5.8 vol % at *z* = 0 mm. This difference is qualitatively displayed in Figure 4a by comparing the intensity of the β peaks indicated for both diffractograms. Moreover, variations in the peak broadening of {200} β—presumably consequence of changes in the domain size as well as strain state of this phase—can be qualitatively observed along *z* in Figure 4c.

Although no evident variations of *a*_β_ are observed for the lower part of the sample between *z* = 0 and 7.75 mm, it is important to note that slight oscillations of ~±0.4 vol % are estimated in this range. These changes can be correlated with those shown in Figure 5a for the intensity evolution of {110} of β, the reflection of this plane presenting—by far—the highest intensity of the entire diffractogram for the β phase.

**Figure 6:**
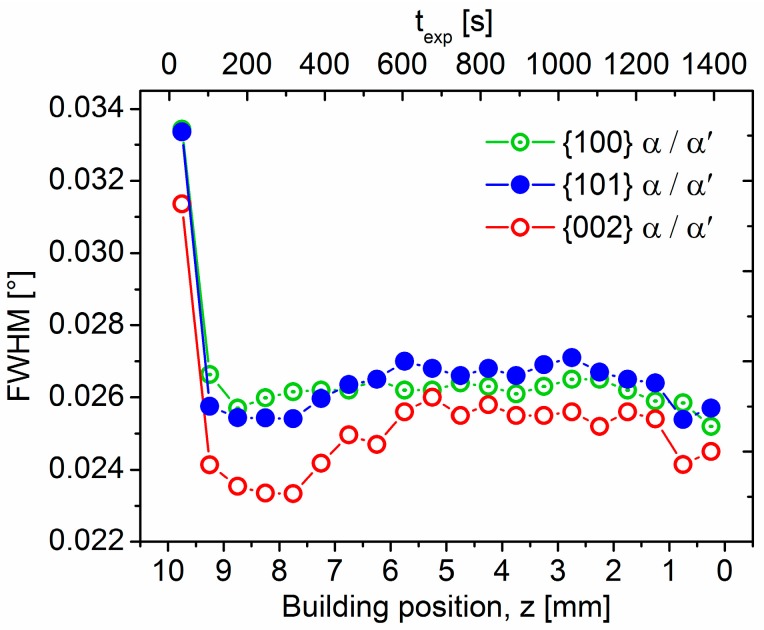
Evolution of the full width at half maximum (FWHM) for hcp {100}, {101} and {002} reflections of α’/α as a function of the building position *z* and exposure time *t_exp_*.

Figure 6 shows the evolution of the FWHM obtained for three representative reflections of α’/α as a function of *z* and *t_exp_*. The results show an abrupt increase of ~30% in the FWHM at building distances from 9.25 up to 9.75 mm, revealing a remarkable widening of the hcp peaks for the region of the SLM Ti-64 alloy mostly formed by martensite. The magnitude of the FWHM variations occurring at lower *z* positions is significantly lower in comparison to this effect, and therefore, they reflect less pronounced modulations of the hcp phase associated with, for example, grain size and/or lattice distortions. Also, a marked anisotropic FWHM evolution can be observed comparing {100} − {101} and {002} α/α’ reflections within *z* = 9.25 − 5.75 mm.

### 3.2. Texture and 3D Architecture of β

**Figure 7:**
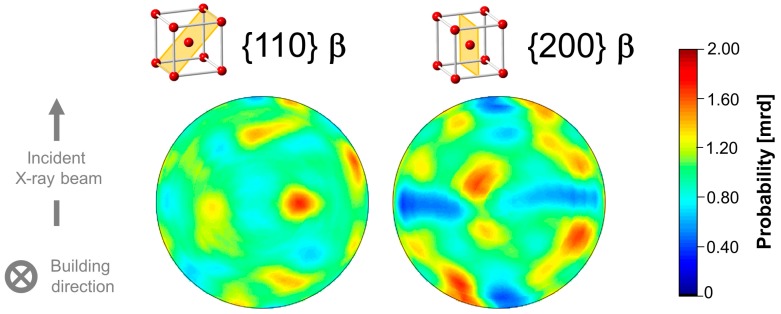
Color-coded pole figures of the crystallographic orientations {110} and {200} of β for the entire Ti-64 SLM sample reconstructed from the sum of the Debye–Scherrer rings acquired by HEXRD along the building direction *z* (*sample length* = 10 mm).

Figure 7 shows color-coded pole figures of the {110} and {200} crystallographic orientations of β calculated for the entire SLM Ti-64 alloy along the building direction (*sample length* = 10 mm). The results reveal that the scanning strategy of the laser leads to a relatively weak texture compared to the strong <100> preferential orientation of β that is typically obtained along the building direction when a more standardized SLM process configuration, i.e., a wider hatch distance, is used (see e.g., [26,27]).

**Figure 8:**
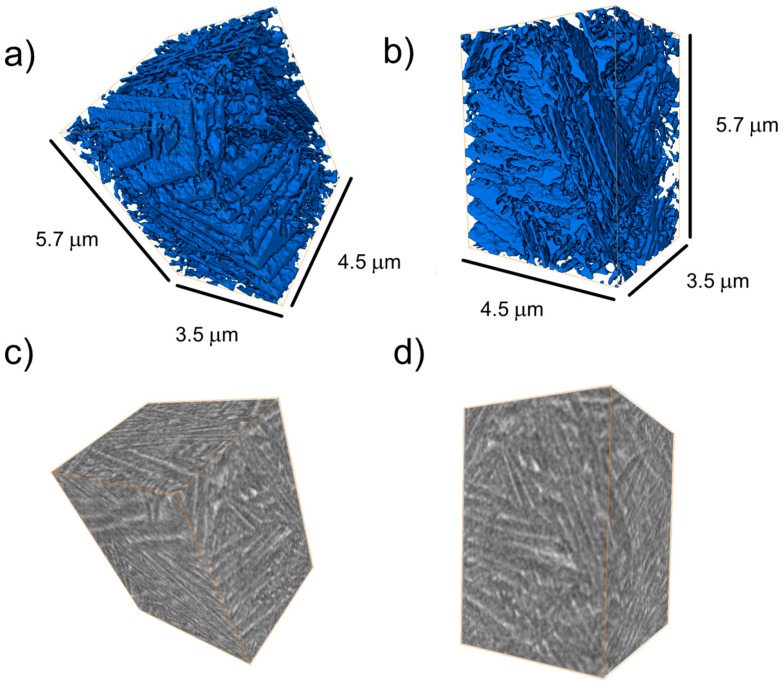
(**a**,**b**) Illustrative perspectives of the interconnected β network (in blue) segmented from (**c**,**d**) its associated holographic X-ray computed tomography (HXCT) reconstruction (voxel size = (10 nm)^3^ and β matrix in bright grey) corresponding to a representative volume of the microstructure formed at the bottom part of the Ti-64 sample (i.e., *z* ~ 0 mm) upon SLM.

HXCT (Figure 8) reveals that—for a region highly affected by the intrinsic SLM heat treatment (i.e., at building position *z* ~ 0 mm)—this phase consists of a thin (≤70–100 nm) and highly interconnected continuous 3D network throughout the investigated volume (blue). Thinner layers of β cannot be resolved by this technique but their presence can be inferred from the TEM results shown in Figure 2a. This indicates that the intrinsic heat treatment applied during SLM induces the formation of a β matrix percolating through the α lamellae. Also, layers of β surrounding α colonies analogous to those observed for Figure 1e can be distinguished in Figure 8a,b.

## 4. Discussion

### 4.1. Effect of the Intrinsic Heat Treatment: Microstructural Evolution along the Building Direction

Usually, the SLM of Ti-64 powder results in an extensive formation of α’ martensite throughout the whole bulk alloy as a consequence of the fast cooling rates achieved during manufacturing (~10^3^–10^8^ K/s [4,5,6,7]). This is the result of the β → α’ transformation—similar to that occurring upon conventional water quenching of the ingot alloy (~10^2^ K/s in relatively thin sections of few mm)—at cooling rates > ~17 K/s [11,28,29]. In the current work, an intensified intrinsic heat treatment (IHT) applied using a relatively tight hatch distance (see Section 3.1), provokes the decomposition of martensite during SLM into a fine lamellar α + β microstructure (α’ → α + β), where the intermetallic phase Ti_3_Al also forms.

#### 4.1.1. Formation of β

The HEXRD investigations presented in Figure 4 and Figure 5 reveal that formation of β occurs in all layers of Ti-64 synthesized using the IHT applied. At *z* positions below the martensitic region of ~ 150–250 µm at the top of the samples, β forms as continuous, thin layers at the interface of α lamellae (see Figure 1d,e and Figure 2a). This is a consequence of the IHT at temperatures at which diffusion is active below the martensite start temperature, *M_s_* (~800 °C for Ti-64 [29]), i.e., T > 200 °C of the building platform. Considering that at *T < M_s_* formation of martensite (β → α’) can be suppressed upon fast cooling, successive precipitation of β takes place instead, owing to the decreasing influence of the effective IHT temperature during further manufacturing. During IHT in the α + β field, segregation and partitioning of V to β, and Al to α, takes place, causing higher stabilities of both phases upon cooling [30]. This can reduce the amount of “retained” α’ martensite during subsequent IHT cycles and the formation of V-enriched β. This results in the formation of α + α’ + β microstructures that evolve during successive series of IHT cycles towards an α + β microstructure. Presumably, preferred sites for V partitioning during IHT cycles are interfaces and also retained β, resulting in the formation of a β-network along the α-interfaces.

The features of lamellar α + β microstructures obtained by ingot metallurgy routes, i.e., the size of α colonies as well as the width of α lamellae, are basically given by continuous cooling after annealing in α + β (usually ~700–850 °C) or β field. For the latter, α transforms from β (β → α + β) through the formation of α at the β grain boundaries followed by the growth of relatively coarse Widmanstätten α colonies [31]. In contrast to this, the alloy undergoes a series of sharp thermal cycles with practically untunable heating and cooling rates generated by the travelling laser during SLM manufacturing [27]. Therefore, the spacing of the α + β lamellar microstructure in the SLM alloy is determined by this “cyclic” heat treatment and can be seen as the result of heat exposure at an effective *T < M_s_* during manufacturing followed by fast cooling.

The higher the effective temperature of the IHT for *T < M_s_*, the higher the amount of β formed in the affected region. This can be observed in Figure 5, where the layers exposed to the highest IHT effective temperature present a local maximum in the volume fraction of β (8.8 vol % for *z* = 9.25 mm) with respect to the significantly smaller, quasi-constant values obtained for *z* ≤ 8 mm (5.8 ± 0.4 vol %). This volume fraction of β at *z* ≤ 8 mm—close to that resulting from metallographic analysis in an IHT-affected Ti-64 SLM alloy (5 vol %) [11]—represents the largest amount of β transformed via IHT and is higher than that obtained for the martensitic region (5.1 vol %).

HXCT (see Figure 8) reveals that for microstructures highly affected by IHT, β layers form as an interconnected 3D network percolating through colonies of α lamellae. Thus, in addition to the general improvement of ductility reported by SLM-produced lamellar microstructures with respect to the martensitic ones (e.g., [11,28]), interconnected lamellar configurations are associated with homogeneous distributions of slip transfer between phases (i.e., across the incoherent α/β interphase), that upon structural loading may lead to greater fatigue resistance, ductility and toughness than martensitic microstructures [29].

LOM shows that during SLM processing, columnar prior β grains form in an inclined manner with respect to the building direction (see Figure 1). The length range obtained in their minor axis direction is similar to that reported previously for Ti-64 SLM alloys (~70–135 µm) [14,26,32]. During SLM, prior β grains usually grow along the building direction due to the vertical heat dissipation front from the melt towards the underlying solid substrate [14,26,32]. In the current study, the solidification process is characterized by an intensified re-melting of material owing to the small hatch distance (Section 3.1). Thus, in addition to vertical heat dissipation, this scanning strategy may promote local heat conduction in the lateral direction towards neighbor solid tracks of the same processing layer. The grain inclination observed correlates with the weak texture obtained, which is far from the typical SLM fiber texture (see Figure 7) and Section 4.2 and e.g., [26,27].

#### 4.1.2. Formation of α

The transition from α’ plates to α lamellae along the building direction of the Ti-64 SLM alloy is reflected in the abrupt decrease of FWHM of the hcp phase observed for the top material layer (*thickness* ~ 150–250 µm) of between *z* = 9.25 and 9.75 mm (see Figure 6). Variations in XRD peak broadening are basically associated with changes in the crystalline domain thickness as well as in the strain condition associated with lattice distortions of the microstructure (e.g., the strain field of dislocations) [33]. These factors are inversely and directly proportional to peak broadening, respectively. Thus, the decrease in FWHM evident in this study indicates a transition from metastable, strained arrangements of thin α’ plates with high internal stresses towards coarser and less strained α lamellae during IHT. Similar variations of peak broadening between α’/α have been reported to occur with decreasing cooling rate for an analogous Ti-64 ingot alloy [34].

The microstructural transition described above correlates with the increase in microhardness towards the top martensitic layer (Figure 3). The values obtained within this region (~425–475 HV 0.05) are very close to those published (~426–479 HV 0.03) for martensitic conditions in Ti-64 SLM produced with comparably larger hatch distances (50–75 µm) [14]. The microhardness variation observed within this α’ range may be caused by the formation of a hierarchical martensitic microstructure of different size distributions of martensite plates (e.g., primary, secondary, tertiary, etc.), which can be generated upon fast cooling from different temperatures during repeated layer deposition (last synthesized layers) [32]. By extrapolating simulated SLM process data for Ti-64 [35,36], a melt pool depth of ~100 µm should be obtained for the laser power used in this study (175 W). Considering that a ~150–250 µm martensitic layer is visible in Figure 1 (region A) it may be assumed that a total depth of ~50–150 µm (difference between melt pool depth and thickness of region A) corresponds to a solid region that undergoes fast cooling from *T > M_s_* resulting in β → α’ martensitic transformation.

On the other hand, the regular microhardness values obtained for the IHT-affected microstructure along the building direction (~420 ± 10 HV 0.2, as shown in Figure 3), are comparable to those reported for fine lamellar α + β microstructures (~439 ± 5 HV 0.5) formed during high-temperature SLM fabrication of the Ti-64 alloy [13].

The α lamellae thickness (*L*_α_) of ~100–400 nm obtained from the tight-hatch scanning strategy implemented in this study can be correlated very well with those obtained using inter-layer times (*t_i_*) of 5 and 8 s, respectively, for Ti-64 SLM [28], namely ~300 ± 100 nm and ~250 ± 50 nm. The *L*_α_ interval obtained couples with that observed after 2 h of post annealing of martensitic Ti-64 SLM samples at 400–600 °C [37]. Therefore, it is plausible to suggest that for the studied alloy and applied SLM conditions, an effective temperature within this range is reached during IHT.

Ultrafine α + β lamellar microstructures such as those produced in the present work can provide attractive combinations of strength, toughness and ductility with associated high yield strength and large elongation to failure values that can reach ~> 1100 MPa and 11.4%, respectively [10,28].

#### 4.1.3. Formation of α_2_-Ti_3_Al

The formation of the intermetallic α_2_-Ti_3_Al phase along the building direction of the Ti-64 sample is revealed using HEXRD by the presence of {101} reflections of this phase shown in Figure 4 as well as by SAD carried out during TEM investigations (Figure 2b). The formation of coherent α_2_ particles in α can increase the yield stress of the alloy but also reduce tensile ductility owing to generation of planar slip bands leading to decrease in fatigue resistance (i.e., easy crack nucleation and propagation) [29].

Precipitation of Ti_3_Al—assumed to be a consequence of Al segregation during rapid cooling—has been suggested for an analogous, fully martensitic SLM alloy [14]. According to previous investigations of Ti-64, the driving force for α_2_ formation in α depends on both the Al concentration and oxygen concentration [38,39]. Thus, the presence of O decreases the solubility limit of Al in α and, consequently, facilitates the precipitation of Ti_3_Al as verified experimentally in Ti-64 alloys containing <0.22 wt % O [38,39]. This amount of O corresponds to the concentration in the Ti-64 alloy investigated (see Section 3.1) and therefore, these results can be correlated here with those obtained using HEXRD and SAD in the current study.

In this work, structural evidence of Ti_3_Al was detected for the α + β lamellar region mostly affected by the IHT. Since precipitation of this phase can occur during aging at 500–600 °C for several hours (e.g., 24 h at 500 °C) [38,39], it is, again, reasonable to conclude that this range of temperatures was reached during IHT for a sufficient transformation time.

### 4.2. Diffusion-Driven Microstructural Stabilization along the Building Direction

Figure 5b shows a steep increase of *a*_β_ towards the lastly deposited region of the Ti-64 SLM samples between *z* ~ 7.75 and 9.75 mm. This effect can be qualitatively correlated with the 2-Theta variations observed in Figure 4c and Figure 5a, and indicates a gradual evolution of the elemental partitioning between β and α’/α as a consequence of the IHT applied inducing martensite decomposition (α’ → α + β) and consequent formation of a fine α + β lamellar microstructure along the building direction (see Section 4.1). During this process, rejection of solute elements from the supersaturated α’ martensite (e.g., Al and V) occurs during thermal treatment in the α + β field (martensite tempering) according to the solubility limit reached during IHT. This results in the formation of α and precipitation of stable β where structural defects such as plate and twin boundaries are. As more and more layers are added, the influence of IHT decreases, the microstructure stabilizes and remains comparatively constant below the martensitic layer.

During the early IHT cycles, the small martensite units may disolve, while fragmentation, coarsening and eventual coalescence of bigger plates may take place. Simultaneously, the interconnected network of β forms (Figure 8). Coalescence and elemental partitioning of single β precipitates leading to the formation of thin layers of β along plate boundaries may occur during IHT [40,41]. The decreasing values in *a*_β_ are presumably associated—as reported in previous works—with a progressive enrichment of β in the concentration of V [39,42]. Thus, these results establish *a*_β_ as an indicator for the microstructural degree of stabilization of SLM Ti-64 along the building direction. The IHT applied in this work provides stabilization of the SLM Ti-64 alloy beyond ~2 mm from the top (*z* = 10 mm) towards the bottom (*z* = 0 mm). This distance can be roughly taken as a height reference of the heat-affected region undergoing elemental diffusion and partitioning below the scanning laser. Consequently, further building beyond *z* > 10 mm would tend to flatten the observed increase of *a*_β_ down to the linearity observed for the already IHT-affected lower building positions.

## 5. Conclusions

In this work, an intensified intrinsic heat treatment was applied during selective laser melting of a Ti-6Al-4V alloy combining optimized processing parameters to minimize porosity with a tight hatch distance associated with long exposure periods at high temperature. The following conclusions can be drawn from the investigations:

- The intensified intrinsic heat treatment applied during SLM provokes extensive martensite decomposition (α’ → α + β) along the building direction. This results in the formation of a uniform, fine lamellar α + β microstructure.
- A relatively thin martensitic layer (~150–250 µm) forms for the lastly synthesized layers of material as a consequence of the IHT influence at *T > M_s_.* The transition from α’ plates to stable α lamellae is reflected along the building direction by an abrupt decrease of the full width at half maximum of hcp reflections as well as by the microhardness evolution.
- The evolution of the lattice parameter of β, *a*_β_, indicates a gradual variation in the IHT-induced element partitioning between β and α’/α along the building direction of the sample. Decreasing *a*_β_ values are presumably associated with a progressive partitioning of V to β. The results obtained establish *a*_β_ as an indicator for the microstructural degree of stabilization of the SLM Ti-6Al-4V alloy.
- Columnar prior β grains form in an inclined manner with respect to the building direction. This may occur due to uneven heat dissipation caused by the chosen scanning strategy and can be correlated with the weak texture obtained for the SLM Ti-6Al-4V alloy.
- In regions highly affected by the IHT, interconnected β layers form as a 3D network percolating through colonies of α lamellae, as revealed by high-resolution synchrotron holographic X-ray computed tomography. Consequently, homogeneous distributions of slip transfer between phases across the incoherent α/β interphase may be obtained.
- Structural evidence of the formation of the intermetallic α_2_-Ti_3_Al phase is revealed by high-energy synchrotron X-ray diffraction along the building direction of the sample as well as by TEM performed at a central region of the samples.

## Figures and Tables

**Table 1:** Main processing parameters used for the selective laser melting (SLM) process.

*P*	*v*	*h*	*F*	*x*	*E_v_* *		
Laser power (W)	Scanning velocity (mm/s)	Hatch distance (µm)	Focal offset distance (mm)	Layer thickness (µm)	Volume energy density (J/mm^3^)	Strategy of laser pattern	Building platform temperature (°C)
175	600	40	2.0	30	243	Zig-zag scanning	200

* $E_{v}=\;\frac{P}{v\cdot h\cdot x}\;\left[J/mm^{3}\right]$.

**Table 2:** Experimental parameters used during the high-energy synchrotron X-ray diffraction (HEXRD) experiments.

Energy (keV)	Wavelength (Å)	Slit-Aperture Size (mm^2^)	Sample-Detector Distance (mm)	Acquisition Time (s)	Read-out Time (s)	Detector
100	0.124	0.5 × 0.5	1601.2	3	2	Perkin Elmer XRD 1621
